# Should we Routinely Reverse Neuromuscular Blockade with Sugammadex in Patients with a History of Heart Transplantation?

**Published:** 2020-01-18

**Authors:** Koichi Yuki, Rebecca Scholl

**Affiliations:** 1 Department of Anesthesiology, Critical Care and Pain Medicine, Cardiac Anesthesia Division, Boston Children’s Hospital, Massachusetts, USA; 2 Department of Anesthesia, Harvard Medical School, Boston, Massachusetts, USA

## Abstract

Cases of cardiac arrest after administration of neostigmine as a neuromuscular reversal agent have been reported in the literature. Sugammadex is a new neuromuscular reversal agent that acts via a different mechanism than acetylcholinesterase inhibitors. Here we reviewed the currently available literature on the use of sugammadex and potential considerations of using sugammadex in patients with a history of heart transplantation. Based on our currently available information, sugammadex administration in heart transplant patients should warrant similar caution and preparation for cardiovascular collapse as acetylcholinesterase inhibitors.

## Introduction

Over 3,000 heart transplantations occur every year in the United States [1]. Following heart transplantation, these patients often undergo cardiac and non-cardiac procedures. General anesthesia with muscle relaxation is often administered in those cases. Many case reports have described cardiac arrest following administration of neostigmine, an acetylcholinesterase inhibitor to reverse neuromuscular blockade. Sugammadex, a relatively new neuromuscular reversal agent, is being used more frequently as an alternative to reversing paralysis. We review the safety of using sugammadex in patients with a heart transplantation.

## Acetylcholinesterase Inhibitor and Cardiac Arrest

Acetylcholinesterase inhibitors such as neostigmine and edrophonium are traditional reversal agents for neuromuscular blockade. Given that the transplanted heart is surgically denervated at the time of harvest, acetylcholinesterase inhibitors were initially expected not to affect heart rate [2]. The study by Bachman, et al., however, challenged this assumption. They studied heart rate response to neostigmine in patients without heart disease, with a history of heart transplantation within 6 months (“recent transplant”) and with a history of transplantation more than 6 months ago (“remote transplant”). They found heart rates were reduced in all of the three groups, with the most sensitivity to neostigmine being no heart disease followed by remote transplant and lastly recent transplant [3,4]. In fact, the transplanted heart gradually gains parasympathetic reinnervation [5], suggesting that neostigmine can reduce heart rate indirectly by increasing acetylcholine levels via its acetylcholinesterase inhibition activity. Reports of severe heart rate reduction leading to asystolic cardiac arrest have been described after neostigmine reversal [6–9]. Interestingly, in all the cases reported so far neostigmine has been the culprit, not edrophonium. Although reinnervation continues to occur following heart transplantation, this is a very slow process and may not be complete even after 15 years [10]. Thus, parasympathetic tone should be greater in normal hearts than in transplanted hearts, and cardiac arrest by acetylcholinesterase inhibitor administration is unlikely to be explained solely by parasympathetic reinnervation. Neostigmine, compared with edrophonium, has a carbamyl group, which directly binds and activates muscarinic acetylcholine receptor [6]. The reported cardiac arrest cases primarily involve patients with existing coronary vasculopathy and a history of rejection, thus patient factors likely also contribute to cardiac arrest susceptibility after neostigmine reversal. The exact cause of cardiac arrest after neostigmine reversal has not been conclusively delineated, but it is reasonable to consider that neostigmine is not a favorable or always safe reversal agent [6].

## Sugammadex as a Solution?

Sugammadex is a modified γ-cyclodextrin that encapsulates the steroidal neuromuscular blockade agents, resulting in a reduction of their free plasma concentrations and termination of muscle relaxation. Sugammadex was first approved in Europe in 2008, and approved by the Food and Drug Administration (FDA) for use in the United States in 2015 [11]. Because this drug does not have anti-cholinesterase activity, there is some motivation to use this drug in patients with a history of heart transplantation [12]. However, “bradycardia or cardiac arrest” is warned as a potential side effect of sugammadex by the company [13]. Thus, it is critical to review the currently available data on sugammadex for use in patients with a history of a heart transplant.

## The incidences of bradycardia and reports of cardiac arrest

Sugammadex is administered as a single bolus injection. For rocuronium and vecuronium, a 4 mg/kg dose is recommended if spontaneous muscle recovery is indicated by a twitch response of 1 to 2 post-tetanic counts (PTC) and there are no twitch responses to train-of-four (TOF) stimulation. A 2 mg/kg dose is advised if spontaneous recovery demonstrates a second twitch in response to TOF stimulation. For rocuronium only, a 16 mg/kg dose is recommended if there is a clinical need to reverse neuromuscular blockade immediately after administration of a single dose of 1.2 mg/kg of rocuronium. In the pooled Phase 1–3 studies [14] that compared the response to 2, 4, or 16 mg/kg of sugammadex in 2914 subjects and 544 subjects in the placebo group, the most common adverse reactions to sugammadex were vomiting, nausea, and headache [15]. Hypotension was seen in 4% of 2 mg/kg group, 5% of 4 mg/kg group and 13% of 16 mg/kg group. In this cohort, bradycardia was seen in 1% of 2 mg/kg group, 1% of 4 mg/kg group and 5% of 16 mg/kg group. Furthermore, several case reports have described extreme bradycardia followed by cardiac arrest after sugammadex administration. A summary of these case reports is shown in Table 1.

Severe cardiac collapse has been reported due to sugammadex-mediated anaphylaxis [15–17], but the cases in Table 1 were considered to occur via a different mechanism. One case report described that sugammadex administration was potentially associated with coronary vasospasm [18]. In this case, a 58-year-old patient, without any history of cardiac disease, underwent cerebral aneurysm clipping surgery and received sugammadex (200 mg) at the end of the case to reverse rocuronium neuromuscular blockade. Hypotension along with ST elevation in lead II was noted, requiring resuscitation. Cardiac catheterization did not show any narrowing of the coronary arteries. This case was not associated with bradycardic arrest. Transient third-degree atrio-ventricular (3^rd^ degree AV) block was reported after a dose of sugammadex [19]. Whether or not 3^rd^ degree AV block and marked bradycardia in Table 1 share a common mechanism is not clear.

The incidence of bradycardia is lower in sugammadex than in neostigmine [20]. Given its lack of cholinergic effects, sugammadex has been used in patients with a history of heart transplantation. Safe use of sugammadex in patients with heart transplantation has been described in case reports, thus it has been proposed to preferentially use sugammadex over neostigmine as a reversal agent [21–23]. However, now we have a case report of cardiac arrest occurring in a patient with a heart transplantation. This was a patient who underwent heart transplantation 3 years prior to this event, previously complicated with rejection. Although the mechanism of bradycardia has not been delineated, its use should raise caution, like neostigmine, and question indiscriminate use of sugammadex in these patients.

## Cyclodextrin properties and potential mechanism of bradycardia

Cyclodextrins are cyclic oligosaccharides derived from starch known to encapsulate lipophilic guest molecules such as steroids [24]. Natural cyclodextrins consist of oligosaccharides containing six (α), seven (β), eight (γ) or more (α−1,4)-linked α-D-glucopyranose units, which have well-defined lipophilic cavity. They are cylindrical with a cage-like structure. α- and β-cyclodextrins have smaller lipophilic cavities (diameter < 6.5 angstroms), while γ-cyclodextrin has the cavity with diameter of 7.5–8.3 angstroms. Rocuronium and vecuronium bind to cyclodextrins with varying affinity, binding most to γ, followed by β and lastly α-cyclodextrins. Sugammadex was designed using γ-cyclodextrin as a prototype, replacing its eight 6-hydroxyl groups with per-6-deoxy-per-6-sulfanyl chains to increase its cavity size. In addition, a carboxyl group was added to the head of this sulfonyl chain. These modifications increased its affinity to rocuronium and vecuronium significantly.

Recognizing that steroids interact with cyclodextrins, the interaction between sugammadex and endogenous steroid hormones has been tested. Levels of endogenous steroidal hormones was affected by sugammadex, indicating their potential interaction with sugammadex, but no adverse effects were reported [25]. Although catecholamines do not have a steroid ring, consisting of a benzene ring with two hydroxyl groups, the interaction between catecholamines and cyclodextrins has been reported. Dopamine bound to cyclodextrins in the following decreasing order β, then α, followed by γ-cyclodextrins [26]. Similarly, both epinephrine and norepinephrine bound to cyclodextrins, β-cyclodextrin more than α-cyclodextrin [27]. The binding of epinephrine and norepinephrine to γ-cyclodextrin was not tested in the study, but we would expect that both would behave like dopamine and bind to γ-cyclodextrin because the molecular size of dopamine is similar to norepinephrine and epinephrine. These observations suggest that sugammadex possibly encapsulates endogenous catecholamines. In fact, bradycardia and hypotension were most often demonstrated in the 16 mg/kg group compared with the 2 mg/kg and 4 mg/kg groups, suggesting sugammadex possibly reduces catecholamine levels, especially at higher doses. Catecholamine levels are known to increase during surgery. Certainly, the potential encapsulation of catecholamines by sugammadex should be tested *in vitro* as well as potential reduction in catecholamine levels by sugammadex should be tested *in vivo* in the future. Other potential mechanisms for the described bradycardia and hypotension with sugammadex administration should be considered as well.

## Practical consideration of sugammadex use in patients with a history of heart transplantation

A number of studies have compared sugammadex and neostigmine in terms of the rate of adverse events such as efficacy of reversal, particularly from moderate and deep neuromuscular block [28], nausea and vomiting [29,30], postoperative bowel movement [31], bradycardia, and anaphylaxis [32]. A meta-analysis found that there were significantly fewer composite adverse events with sugammadex induced reversal of neuromuscular blockade including postoperative nausea and vomiting, postoperative residual paralysis and bradycardia [33]. The recovery of postoperative bowel movement was also faster in sugammadex [31]. However, the incidence of laboratory confirmed anaphylaxis was noted only in sugammadex group with an incidence of 0.02% in the Japanese series [32]. This was very comparable to the incidence (0.024%) of anaphylaxis noted in the post-marketing surveillance in the United States. Additionally, laryngospasm and bronchospasm events after sugammadex administration have been reported in the literature [34–36], suggesting that the use of sugammadex is not without risk.

A study examining the safety of sugammadex in 116 adult cardiac patients (NYHA class II and III) did not show adverse effects [37]. In a study comparing neostigmine and sugammadex in a total of 90 adult cardiac patients (NYHA class II and III), the sugammadex reversal cohort demonstrated lower heart rate and blood pressure than the neostigmine group [38]. To date, there is limited available data describing the safety profile of using sugammadex in patients with heart transplants. Although the mechanism of sugammadex-induced bradycardia is not known, sugammadex can be equally problematic in both denervated and innervated hearts if it affects catecholamine levels, as described above. As suggested in cardiac arrest cases associated with neostigmine, patient factors could contribute a significant role. The physiology of a transplanted heart can be manifested as restrictive physiology with elevated filling pressures, increased end-diastolic and end-systolic volumes, and low normal left ventricular ejection fraction [39]. Therefore, cardiac output can be quite dependent on heart rate. A relative reduction in heart rate, in this physiologic state, could have a more detrimental consequence than in a normal heart. Until we have more information, we may not have a clear consensus on the superiority of muscle relaxant reversal drugs. It is safe to recommend that direct agonists, such as epinephrine, should be immediately available when providers must reverse muscle paralysis with either neostigmine or sugammadex in patients with heart transplantation.

## Conclusions

Although sugammadex is devoid of cholinergic effects, it can still result in cardiovascular instability. Indiscriminate administration should be avoided. Thus, sugammadex administration in heart transplant patients should warrant similar caution and preparation for hemodynamic changes and possibly collapse as neostigmine.

## Figures and Tables

**Table 1.** Cases of bradycardia cardiac arrest.

Patients	Events	References
10-year-old, 21-Kg, history of heart transplant (3 years ago) for hemodynamic catheterization and endomyocardial biopsy	Muscle relaxed with rocuronium. TOF of 3/4 prior to reversal with sugammadex (2 mg/kg). HR dropped from 102/min to 26/min. Epinephrine (2 mcg/kg) and chest compression for 10–15 seconds. HR increased to 160/min.	King, et al. [40]
76-year-old, 65-Kg for radical prostatectomy	Muscle relaxed with rocuronium. TOF of 2/4 prior to reversal with sugammadex (130 mg). In 2 minutes, HR dropped to 40s along with PVC. Ephedrine 10 mg was given but HR further dropped to < 20/min. Chest compression was initiated, and atropine 0.5 mg was given with return of spontaneous circulation. However, this patient experienced two more episodes of cardiac arrest, both of which were managed with chest compression and epinephrine bolus, and vasoactive agent infusion with good effect.	Ko, et al. [40]
54-year-old, 96-Kg, a history of hypertension and obesity for emergent umbilical herniorrhaphy	Muscle relaxed with rocuronium. TOF of 2/4 prior to reversal with sugammadex (2 mg/kg). Within 30 sec, HR reduced to 30/min, then asystole followed. Atropine 1 mg was given with good effect.	Oliveira, et al. [42]
60-year-old, 82-Kg for prostatectomy	Muscle relaxed with rocuronium. TOF of 4/4 prior to reversal with sugammadex (2.4 mg/kg). Within one minute, HR dropped from 75–80/min to 35/min. Although atropine 1 mg was given, cardiac arrest ensued. Chest compression was initiated, and total of 7 mg epinephrine and 1 gm calcium were given, with return of spontaneous circulation.	Sanoja, et al. [42]
41-year-old, 72-Kg, lung cancer for gastroduodenoscopy	Muscle relaxed with rocuronium. TOF of 2/4 prior to reversal with sugammadex (300 mg). Within 2 min, HR dropped to 25/min with no palpable pulse. Chest compression and epinephrine 1 mg with return of circulation.	Bhavani [43]
60-year-old, 88-Kg, with a history of cerebrovascular accident, asthma, hypertension, hypothyroidism, chronic kidney disease for endoscopic submucosal resection of a lesion in the stomach	Muscle relaxed with rocuronium. TOF of 4/4 prior to reversal with sugammadex (200 mg). The patient was extubated. One minute later, HR dropped to low 30s, and progressed into asystole. Chest compression and epinephrine (30 μg) were given with return of spontaneous circulation.	Bhavani [43]
